# Knockdown of lncRNA *MIR31HG* inhibits adipocyte differentiation of human adipose-derived stem cells *via* histone modification of *FABP4*

**DOI:** 10.1038/s41598-017-08131-6

**Published:** 2017-08-14

**Authors:** Yiping Huang, Chanyuan Jin, Yunfei Zheng, Xiaobei Li, Shan Zhang, Yixin Zhang, Lingfei Jia, Weiran Li

**Affiliations:** 1 Department of Orthodontics, Peking University School and Hospital of Stomatology, Beijing, 100081 China; 2 Department of Prosthodontics, Peking University School and Hospital of Stomatology, Beijing, 100081 China; 3 Central Laboratory, Peking University School and Hospital of Stomatology, Beijing, 100081 China; 4 National Engineering Laboratory for Digital and Material Technology of Stomatology, Beijing Key Laboratory of Digital Stomatology, Beijing, 100081 China

## Abstract

Adipogenesis plays an important role in the regulation of whole-body energy homeostasis and is inextricably related to obesity. Several studies have highlighted the relevance of microRNAs in adipocyte differentiation, but the contributions of long non-coding RNAs (lncRNAs) are still largely uncharacterized. Here, we determined that lncRNA *MIR31HG* is related to adipocyte lineage commitment. We demonstrated that knockdown of *MIR31HG* inhibited adipocyte differentiation, whereas overexpression of *MIR31HG* promoted adipogenesis *in vitro* and *in vivo*. Furthermore, inhibition of *MIR31HG* reduced the enrichment of active histone markers, histone H3 lysine 4 trimethylation (H3K4me3) and acetylation (AcH3), in the promoter of the adipogenic-related gene, fatty acid binding protein 4 (*FABP4*), leading to suppression of its expression and adipogenesis. These results provide new insights into the molecular mechanisms of *MIR31HG* in terms of adipogenesis and may have implications for obesity and associated disorders.

## Introduction

Obesity is an important factor in various diseases, particularly heart disease, diabetes, hypertension, and cancer^1, 2^. The global prevalence of obesity has greatly increased and constitutes a public health crisis^3^. Adipogenesis plays key roles in the regulation of whole-body energy homeostasis and is inextricably related to obesity. It is a complicated process, involving the proliferation of precursor cells, their commitment to the adipogenic lineage, and terminal differentiation^4^. A clearer understanding of the molecular mechanisms that initiate differentiation of stem cells into adipocytes would facilitate the development of methods for the treatment of obesity and other adipogenic-differentiation-related disorders. Adipose-derived stem cells (ASCs) are a ready and ideal cell model for human adipogenesis. The ability of ASCs to differentiate into mature adipocytes has been demonstrated^5, 6^.

Recently, emerging studies of non-protein-coding RNAs (ncRNAs) in adipocyte commitment provide new insights into the molecular basis of adiposity. A class of small ncRNAs, including *miR-25* ^7^, *miR-29b* ^8^, *miR-93* ^9^, *miR-148a* ^10^, and *miR-1275* ^11^, have been identified as important regulators of adipogenic differentiation by targeting various genes involved in cell self-renewal and differentiation. In contrast, the functional contributions of long non-coding RNAs (lncRNAs), tentatively defined as ncRNAs > 200 nucleotides in length^12, 13^, in ASC adipogenic differentiation remain largely unknown. A recent study has profiled the transcriptome of mature adipocytes and pre-adipocytes and identified 175 lncRNAs that are differentially expressed during adipogenesis^14^. However, only a few lncRNAs, such as *ADNCR* ^15^, *ADINR* ^16^, *PU.1-as* ^17^, and *NEAT1* ^18^, have been shown to participate in the genetic control of adipogenic differentiation of ASCs or pre-adipocytes. The importance and potential roles of most lncRNAs in adipocyte lineage commitment remain uncharacterized.

The miR-31 host gene (*MIR31HG*, GenBank accession number, NR_027954), located on chromosome 9 (9p21.3), is transcribed into an lncRNA^19^. *MIR31HG* has been reported to affect cell proliferation in various cancers^19–22^, and our previous study shows that it can affect osteoblast differentiation^23^, indicating an important biological function of *MIR31HG* in cellular growth and differentiation. In this study, we determined the role of *MIR31HG* in adipocyte differentiation. We found that knockdown of *MIR31HG* inhibited adipogenesis of human ASCs (hASCs) *in vitro* and *in vivo*, whereas overexpression of *MIR31HG* promoted this process. In mechanism, *MIR31HG* participated in histone methylation and acetylation of fatty acid binding protein 4 (*FABP4*). These results reveal new insights into the mechanisms of adipogenic differentiation and provide a potential molecular target for the treatment of obesity and related diseases.

## Results

### LncRNA *MIR31HG* is involved in adipocyte differentiation of hASCs

**Figure 1:**
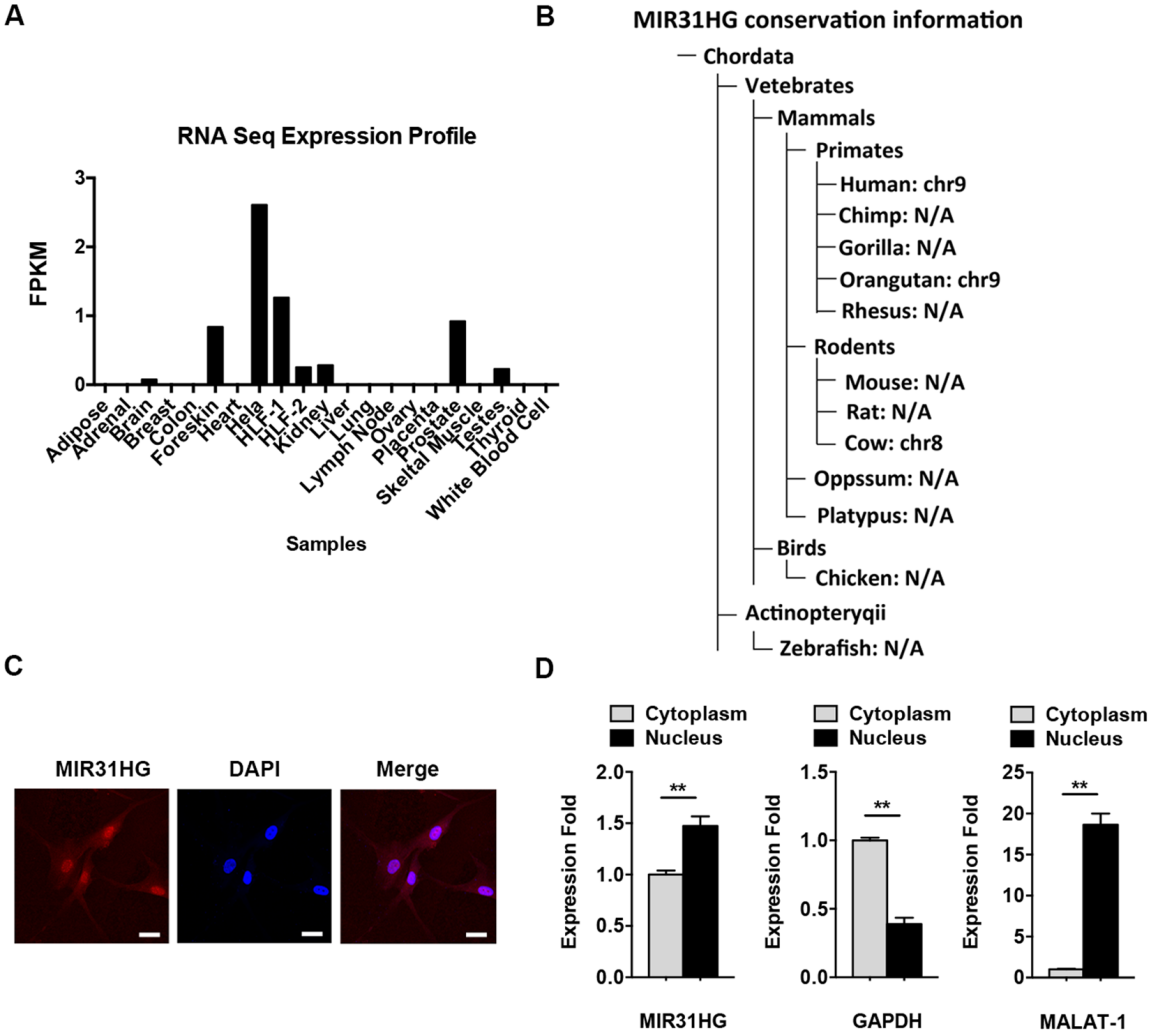
Characterization of lncRNA *MIR31HG*. (**A**) Expression profile of *MIR31HG* in human tissues. FPKM: fragments per kilobase of transcript per million fragments mapped. (**B**) Conservation information of lncRNA *MIR31HG*. Chr: chromosome. (**C**) Confocal FISH images showing localization of *MIR31HG* in hASCs. Scale bars: 20 μm. (**D**) The expression of *MIR31HG*, *GAPDH*, and *MALAT-1* in nuclear or cytoplasmic fraction of hASCs. *GAPDH* and *MALAT-1* were set as controls. Results are presented as mean ± SD (**P < 0.01).

To gain an understanding of *MIR31HG*, we firstly studied the expression pattern of *MIR31HG* in human tissues. RNA-seq data from NONCODE (http://www.noncode.org) indicated that *MIR31HG* is expressed in a number of tissues or cell lines, such as foreskin, kidney, prostate, testes, hela cells, etc (Fig. 1A). Conservation information showed that *MIR31HG* is expressed in several mammals, including human, orangutan, and cow, but absent in other chordates (Fig. 1B). Single-molecule RNA fluorescence *in situ* hybridization of *MIR31HG* showed that the transcript was localized both in the nucleus and cytoplasm of hASCs, predominantly in the nucleus (Fig. 1C). Its localization was also confirmed by nuclear/cytoplasm fractionation (Fig. 1D).

**Figure 2:**
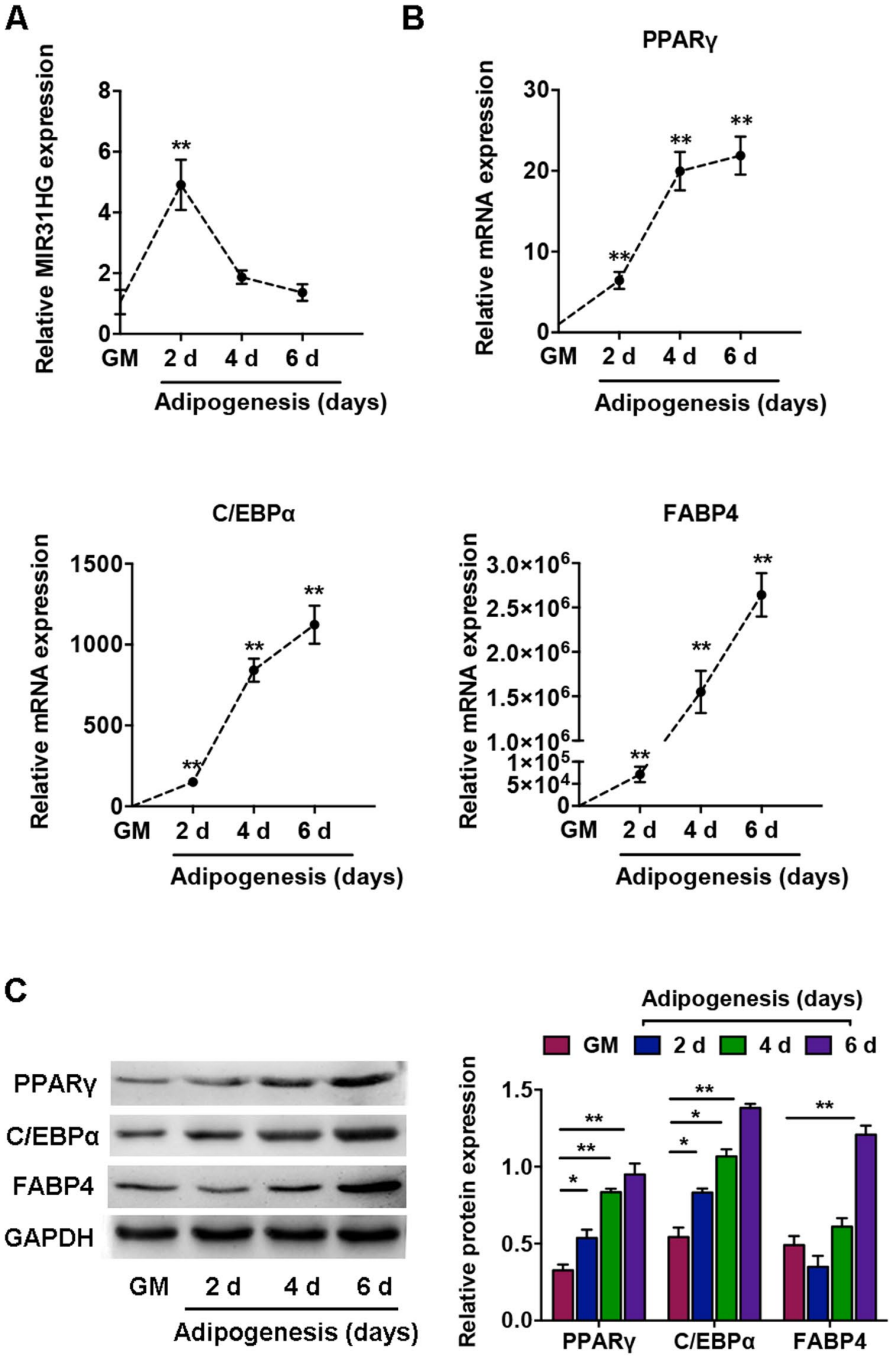
Dynamic Expression profile of *MIR31HG* during adipogenic differentiation of hASCs. (**A**) Relative expression of *MIR31HG* on day 2, 4, and 6 during differentiation as determined by qRT-PCR analysis normalized to the undifferentiation group cultured in growth medium (GM). (**B**) Relative mRNA expression levels of the adipogenic markers *PPARγ*, *C/EBPα*, and *FABP4* at the indicated time points, as in (**A**). Gene expression plotted as fold-change relative to the GM group. (**C**) Western blot analysis (left) and quantification (right) of protein expression of PPARγ, C/EBPα, FABP4, and the internal control GAPDH at the indicated time points, as in (**A**). Results are presented as mean ± SD (*P < 0.05, **P < 0.01 compared to the undifferentiated hASCs).

To study the functional role of *MIR31HG* during adipogenesis, we analyzed *MIR31HG* expression profile during adipocyte differentiation of hASCs. *MIR31HG* exhibited significantly increased expression after 2 days compared to undifferentiated hASCs and returned to normal levels after 4 days (Fig. 2A). The expression of the genes associated with adipogenic differentiation, peroxisome proliferator-activated receptors -γ (*PPARγ*), CCAAT/enhancer-binding protein α (*C/EBPα*), and *FABP4*, was substantially upregulated after adipogenic differentiation induction (Fig. 2B and C).

### *MIR31HG* promotes cell proliferation

**Figure 3:**
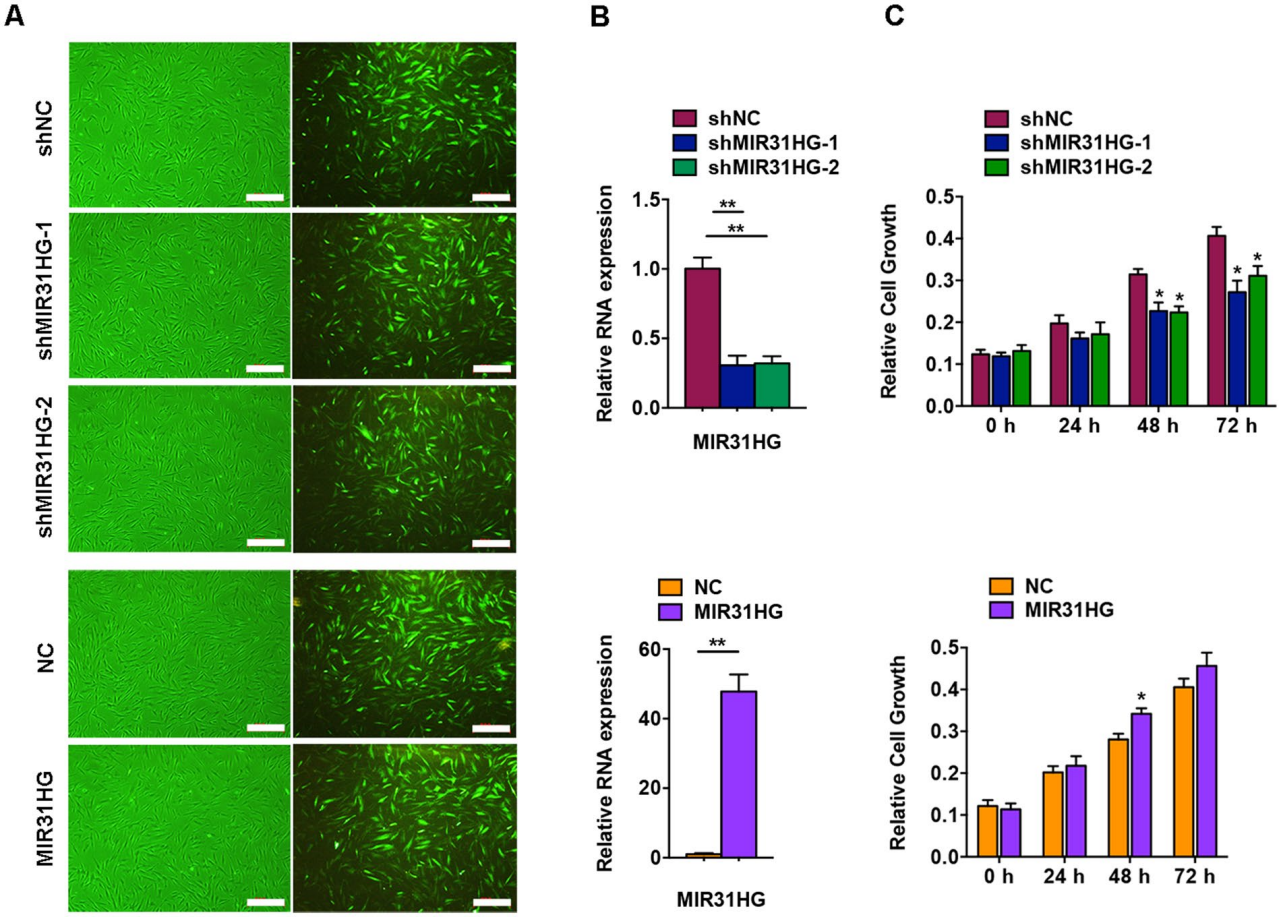
*MIR31HG* promoted cell proliferation of hASCs. (**A**) The hASCs were transfected with lentivirus expressing sh*MIR31HG-1*, sh*MIR31HG-2*, the scrambled nontargeting vector (shNC), *MIR31HG*, or the scrambled vector (NC). Fluorescent photomicrographs show the efficiency of lentivirus transduction (>90%). Scale bar, 500 μm. (**B**) Relative expression of *MIR31HG* in the sh*MIR31HG* groups (up) or the *MIR31HG* group (down). (**C**) CCK-8 assay showing cell proliferation in the sh*MIR31HG* groups (up) or the *MIR31HG* group (down). Results are presented as mean ± SD (*P < 0.05, **P < 0.01).

To further study the biological function of *MIR31HG*, we stably overexpressed and knocked down *MIR31HG* in hASCs using lentivirus transfection. Two different shRNA sequences targeting *MIR31HG* were designed to control for potential off-target shRNA effects. The transduced hASCs did not exhibit morphological changes compared with the control. The efficiency of lentiviral transduction was >90% (Fig. 3A), and the expression of *MIR31HG* was significantly reduced by ~70% in the knockdown group and increased more than 50-fold in the overexpressed group (Fig. 3B).

Since *MIR31HG* expression was induced at an early stage of adipogenic differentiation, we determined the biological function of *MIR31HG* in cell proliferation. According to the CCK-8 assay results, we found that *MIR31HG* overexpression slightly promoted cell proliferation, while *MIR31HG* knockdown decreased cell growth by ~30% relative to negative control at 48 h and 72 h in hASCs (Fig. 3C).

### Knockdown of *MIR31HG* inhibits adipocyte differentiation

**Figure 4:**
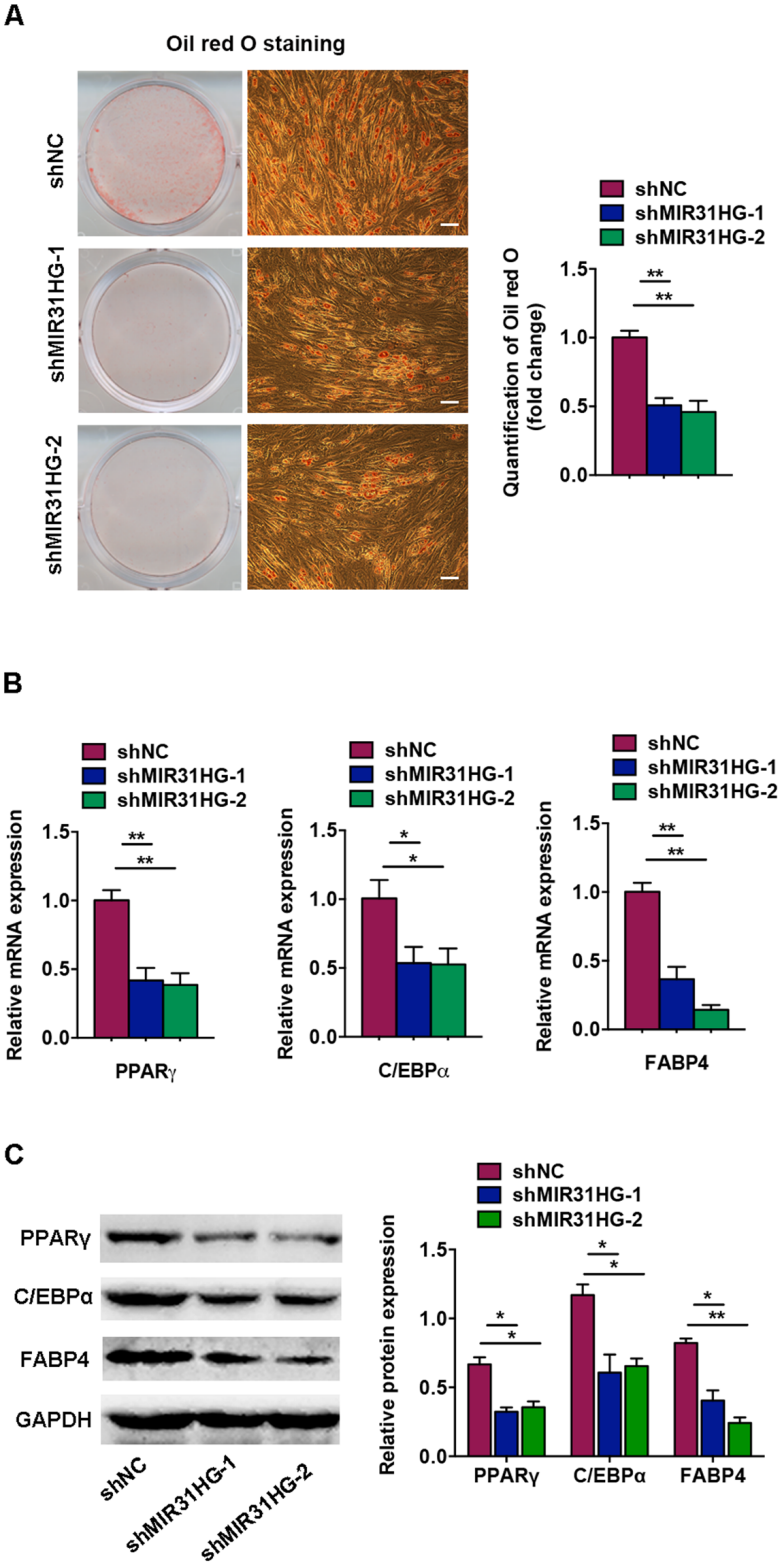
Knockdown of *MIR31HG* inhibited adipogenic differentiation. The hASCs were transfected with lentivirus expressing sh*MIR31HG-1*, sh*MIR31HG-2*, or the scrambled nontargeting vector (shNC). (**A**) Images of Oil red O staining on day 8 of adipocyte differentiation. Scale bar, 100 μm. Histograms show quantification of Oil red O staining by spectrophotometry (normalized to shNC group). (**B**) Relative mRNA expression of the adipogenic factors *PPARγ*, *C/EBPα*, and *FABP4* measured by qRT-PCR on day 4 of adipogenic induction. (**C**) Western blot analysis of PPARγ, C/EBPα, FABP4, and GAPDH on day 4 of adipogenic induction. Histograms show quantification of the band intensities. Results are presented as mean ± SD (*P < 0.05, **P < 0.01).

We then investigated the impact of *MIR31HG* on adipogenic differentiation. We stably knocked down endogenous *MIR31HG* in hASCs. After induction to the adipogenic lineage, *MIR31HG* knockdown significantly reduced adipocyte numbers, as indicated by Oil red O staining on day 8 of differentiation (Fig. 4A). Accordingly, the mRNA levels of the adipocyte-specific markers, *PPARγ*, *C/EBPα*, and *FABP4*, were substantially inhibited by the depletion of *MIR31HG* (Fig. 4B). Consistently, the protein levels of PPARγ, C/EBPα, and FABP4 were downregulated in hASCs with *MIR31HG* knockdown on day 4 of adipogenic induction (Fig. 4C).

### Overexpression of *MIR31HG* promotes adipocyte differentiation

**Figure 5:**
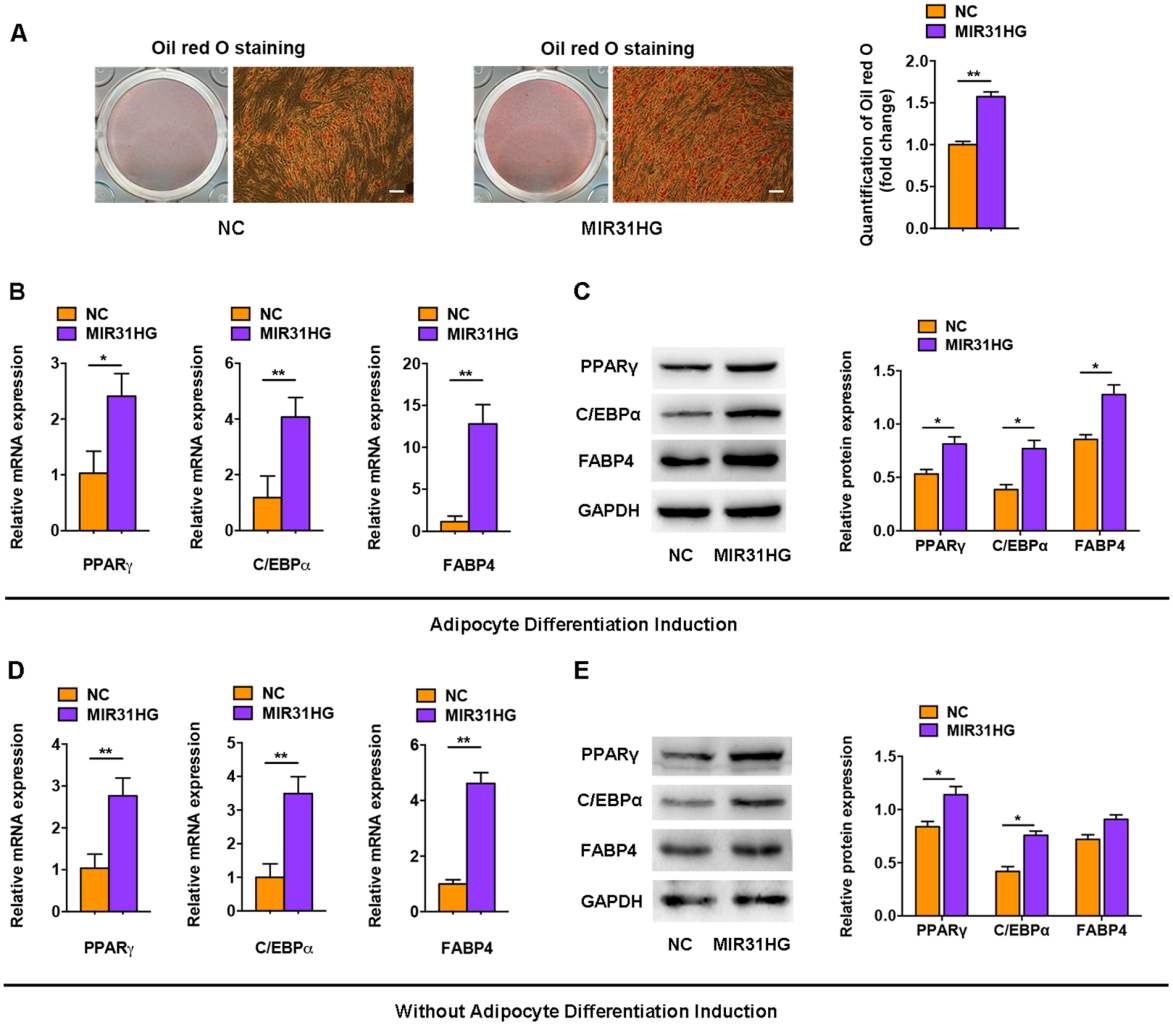
*MIR31HG* promoted adipogenic differentiation. The hASCs were transfected with lentivirus expressing *MIR31HG* or the scrambled vector (NC). (**A**) Images of Oil red O staining on day 8 of adipocyte differentiation. Scale bar, 100 μm. Histograms show quantification of Oil red O staining by spectrophotometry (normalized to NC group). (**B**) Relative mRNA expression of the adipogenic genes *PPARγ*, *C/EBPα*, and *FABP4* on day 4 of differentiation. (**C**) Western blot analysis (left) and quantification (right) of protein expression of PPARγ, C/EBPα, FABP4, and GAPDH on day 4 of differentiation. (**D**) Relative mRNA expression of the adipogenic genes *PPARγ*, *C/EBPα*, and *FABP4* without adipogenic differentiation. (**E**) Western blot analysis (left) and quantification (right) of protein expression of PPARγ, C/EBPα, FABP4, and GAPDH without adipogenic differentiation. Data are presented as mean ± SD (*P < 0.05, **P < 0.01).

We stably overexpressed *MIR31HG* in hASCs. In the presence of adipogenic agents, *MIR31HG* transfection into hASCs led to a significant increase in adipocyte formation, as shown by Oil red O staining on day 8 of differentiation (Fig. 5A). Consistently, *MIR31HG* substantially increased the mRNA (Fig. 5B) and protein (Fig. 5C) levels of the adipogenic genes, *PPARγ*, *C/EBPα*, and *FABP4* on day 4 of adipogenic differentiation. Interestingly, we found that even without adipogenic treatment, supplementation with *MIR31HG* alone increased the expression of adipogenic factors, *PPARγ*, *C/EBPα*, and *FABP4*, as examined by qRT-PCR (Fig. 5D) and Western blot (Fig. 5E). However, *MIR31HG* alone was not sufficient to induce intracellular lipid accumulation without adipogenic supplements as indicated by Oil red O staining (Supplementary Figure 1).

### Histone modification of the *FABP4* promoter region by *MIR31HG*

**Figure 6:**
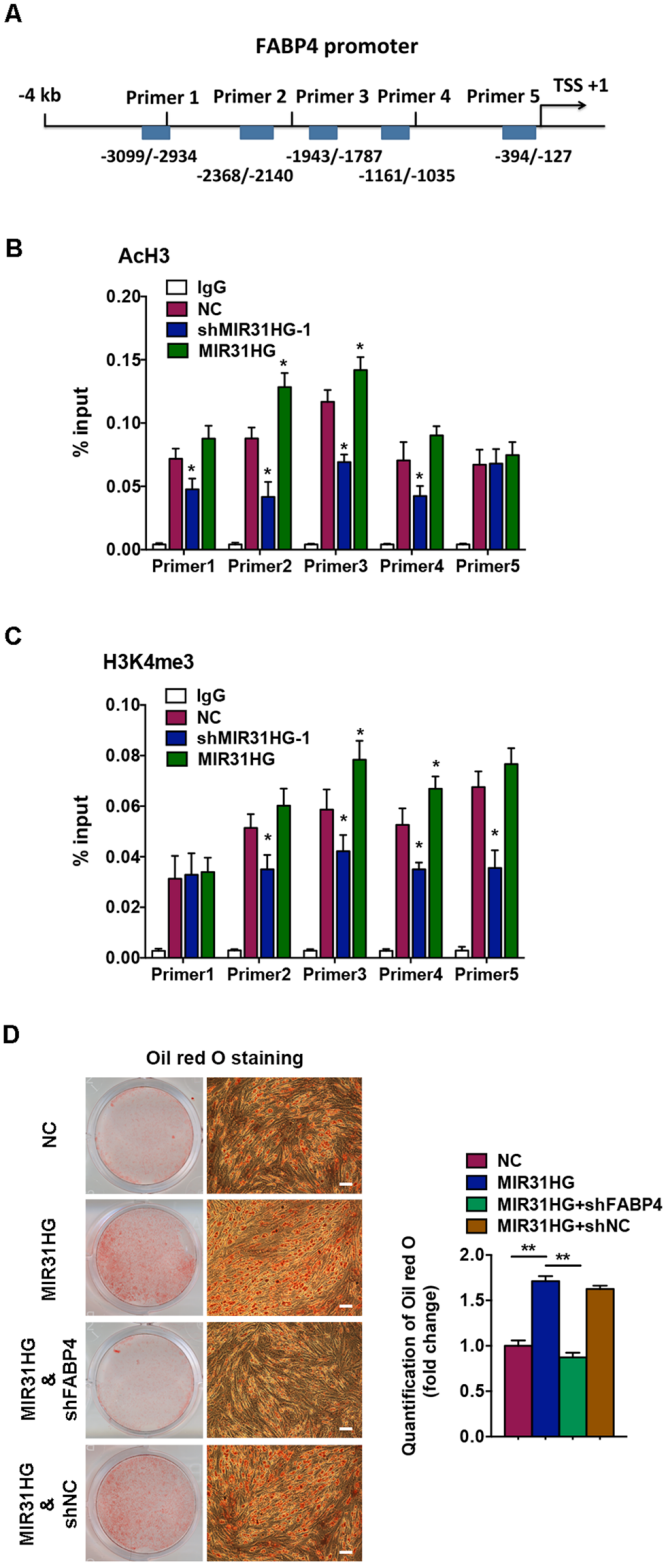
Inhibition of *MIR31HG* reduced the levels of AcH3 and H3K4me3 in the *FABP4* promoter. (**A**) Diagram of the *FABP4* promoter and location of the primers. Positions marked are relative to the transcriptional start site (TSS). (**B**) ChIP assay of AcH3 in the *FABP4* locus in hASCs with *MIR31HG* knockdown (sh*MIR31HG*-1), *MIR31HG* overexpression (*MIR31HG*), or the scrambled vector (NC). Quantitative PCR data of each group were normalized to its input as % input. IgG served as negative control. (**C**) ChIP assay of H3K4me3 in the *FABP4* locus in hASCs with *MIR31HG* knockdown (sh*MIR31HG*-1), *MIR31HG* overexpression (*MIR31HG*), or the scrambled vector (NC). Quantitative PCR data of each group were normalized to its input as % input. IgG served as negative control. (**D**) Images of Oil red O staining on day 8 of adipogenic differentiation in hASCs with *MIR31HG* overexpression (*MIR31HG*) with or without *FABP4* knockdown (sh*FABP4*). Histograms show quantification of Oil red O staining by spectrophotometry (normalized to NC group). Data are presented as mean ± SD (*P < 0.05).

One way that lncRNAs acquire functionality is by interacting with heteronuclear proteins or chromatin modification complexes^24^. We determined the effect of *MIR31HG* on histone modification of adipogenic transcription factors. Combined the results of qRT-PCR and Western blot analysis, we found that *MIR31HG* had significant effect on *FABP4* (Figs 4 and 5), we thus examined local histone H3 acetylation and methylation in chromatin associated with the *FABP4* promoter region using chromatin immunoprecipitation (ChIP) assay. The histone-associated DNAs, immunoprecipitated with antibodies against AcH3 and H3K4me3, were individually amplified with five primer sets covering the *FABP4* promoter region (Fig. 6A). The results showed the marked differences in the levels of histone H3 acetylation and methylation in *MIR31HG* knockdown or overexpressed cells compared to the control group. The level of AcH3 was decreased in *MIR31HG* knockdown cells and increased in *MIR31HG* overexpression cells in the region containing primers 1–4 (Fig. 6B), while the level of H3K4me3 was decreased in *MIR31HG* knockdown cells and increased in *MIR31HG* overexpression cells in the fragment containing primers 2–5 (Fig. 6C).

### Knockdown of *FABP4* abolishes the pro-adipogenic effect of *MIR31HG*

To determine whether *MIR31HG* regulates adipogenesis via histone modification of *FABP4* gene, we knocked down *FABP4* expression in hASCs with *MIR31HG* stably overexpression. After adipogenic induction, knockdown of *FABP4* abolished the promotion of adipogenesis induced by *MIR31HG*, as indicated by Oil red O staining on day 8 of differentiation (Fig. 6D).

### *MIR31HG* promotes newly formed adipose tissue *in vivo*

**Figure 7:**
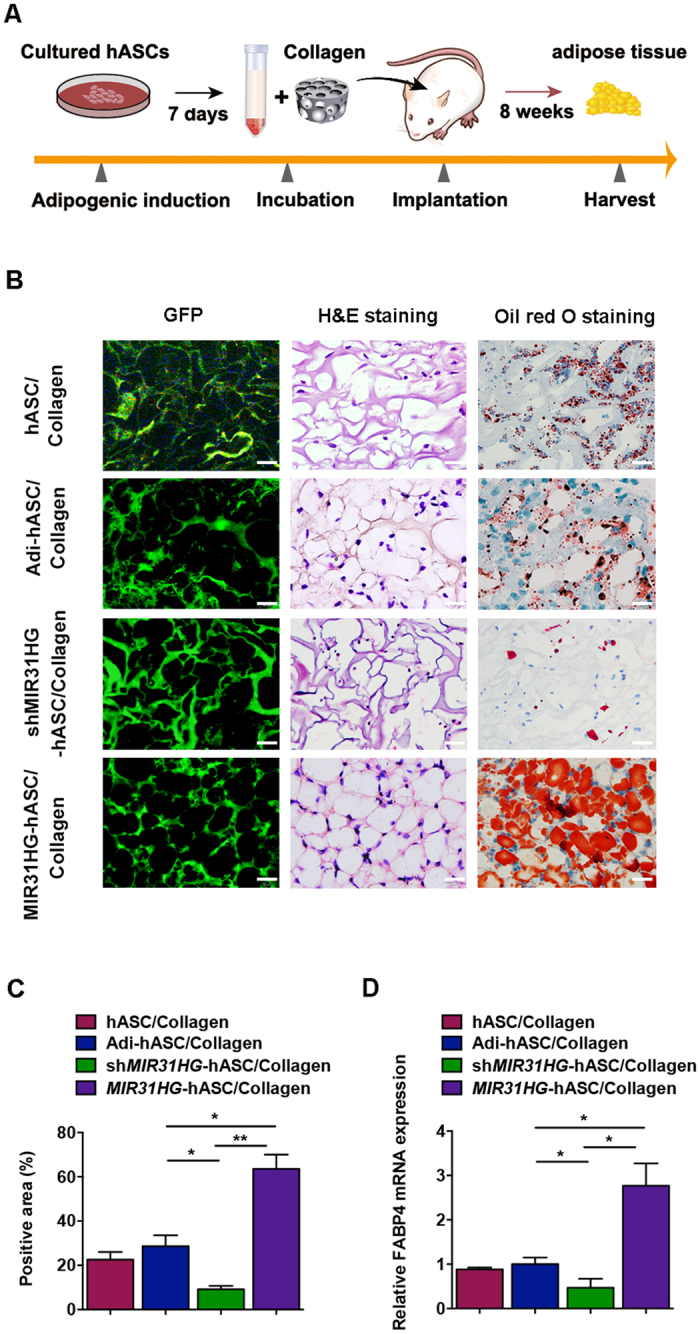
*MIR31HG* knockdown inhibited adipose tissue formation *in vivo*. (**A**) Schematic diagram illustrating the experimental setup. (**B**) Fluorescent images, H&E staining, and Oil red O staining of the specimens in hASC/Collagen group (implanted with non-induced hASC/Collagen complex), Adi-hASC/Collagen group (implanted with adipogenic-induced hASC/Collagen complex), sh*MIR31HG*-hASC/Collagen group (implanted with adipogenic-induced sh*MIR31HG*-hASC/Collagen complex), and *MIR31HG*-hASC/Collagen group (implanted with adipogenic-induced *MIR31HG*-hASC/Collagen complex). Scale bar, 25 μm. (**C**) Quantitative analysis of the Oil red O-stained areas of newly generated tissue at 8 weeks after implantation. The areas were expressed as percentages of the total areas. (**D**) Relative mRNA expression of *FABP4* in the newly generated tissue from indicated groups (normalized to Adi-hASC/Collagen group). Data are presented as mean ± SD (*P < 0.05, **P < 0.01).

To determine the role of *MIR31HG in vivo*, we subcutaneously implanted hASCs stably expressing *MIR31HG*, sh*MIR31HG*-1, or control mixed with a collagen scaffold in the dorsum of nude mice (Fig. 7A). The neo-generated tissue was histologically composed of numerous lobule-like structures separated from each other by fibrous tissue 8 weeks after implantation. The progressive development of adipose tissue was further characterized by Oil red O staining, showing intracellular lipid accumulation. The green fluorescent protein (GFP) -positive stem cells were tracked using fluorescence microscope. The *MIR31HG* group had more adipose-tissue-like constructs stained positively for Oil red O, while the sh*MIR31HG* group had fewer lipid droplets stained for Oil red O (Fig. 7B). Quantification of the newly generated adipose tissue consistently showed a greater ratio of adipogenesis in the *MIR31HG* group (63.5%) and a lower ratio in the sh*MIR31HG* group (9.2%) compared to control group (28.6%) (Fig. 7C). The qRT-PCR analysis of total RNA from the newly formed tissues showed that the *FABP4* expression was reduced in the sh*MIR31HG* group, while increased in the *MIR31HG* group (Fig. 7D).

## Discussion

In this study, we demonstrated that knockdown of *MIR31HG* inhibited the adipocyte differentiation of hASCs, whereas overexpression of *MIR31HG* promoted the commitment of adipocytes. *MIR31HG* can also affect cell proliferation of hASCs, consistent with several studies of *MIR31HG* in carcinogenesis^19–22^. Moreover, our previous study shows that inhibition of *MIR31HG* expression promotes osteogenesis^23^. Taken together, *MIR31HG* may be involved in balancing self-renewal and stem cell differentiation. Interestingly, a previous study has subcutaneously injected recombinant adenovirus expressing *Adamts1* shRNA adjacent to the inguinal fat pad in a preclinical mouse model, and found that the mice show enlargement of inguinal fat pads, greater weight, and impaired insulin sensitivity and glucose tolerance compared to control mice^25^. Our study indicating the role of *MIR31HG* in adipocyte commitment may also provide a potential target for the treatment of obesity through subcutaneous injection of sh*MIR31HG*. On the other hand, engineering adipose tissue is an alternative to plastic and reconstructive surgery for restoring body contours in patients who have lost soft tissue because of external injury, congenital malformation, or surgical resection^5, 6^. Supplementation of *MIR31HG* in hASCs combined with a degradable scaffold may serve as a promising approach to improve adipogenesis in adipose tissue engineering.

*MIR31HG* contributed to histone H3 methylation and acetylation of the *FABP4* gene. Histone modifications are significant reversible mechanisms of epigenetic regulation of gene expression. Dramatic changes in epigenetic signatures have been observed during adipogenesis^26–28^. A previous study has profiled the dynamic histone modifications of key adipogenesis regulatory genes during adipocyte commitment, and found that both H3 acetylation and K4 trimethylation levels increase in all the induced adipogenic genes and correlate positively with gene activity^27^. The most obvious increase is observed in the *FABP4* gene^27^. This study supports our results showing that knockdown of *MIR31HG* reduced the enrichment of AcH3 and H3K4me3 in the *FABP4* promoter, leading to suppression of *FABP4* expression. And, knockdown of *FABP4* expression abolished the effect of *MIR31HG* in adipogenesis. Adipocyte differentiation is a highly regulated process governed by *PPARγ* ^29, 30^, which cooperates with *C/EBP* to induce the expression of many genes important for terminal differentiation, such as *FABP4* and *CD36* ^31^. *FABP4* is highly expressed in adipose tissue and is able to bind and transport fatty acids and other lipophilic compounds^32^. In mouse models, *FABP4* deficiency partially protects mice against the development of insulin resistance associated with genetic or diet-induced obesity^33, 34^. Previous studies suggest that targeting *FABP4* with inhibitors is possible to prevent and treat metabolic diseases, such as type II diabetes and atherosclerosis^35^. Thus, *MIR31HG* may serve as a molecular target for metabolic diseases at least partially through suppression of *FABP4*. However, during the adipocyte differentiation, the expression of *MIR31HG* was transiently upregulated in the early stage (day 2) and returned to normal levels in the late stage (day 6). This expression pattern did not correlate with the expression pattern of adipogenic markers. Many different events contribute to adipocyte determination and differentiation including the coordination of a complex network of transcription factors, cofactors and signaling intermediates^4^. We cannot exclude the possibility that other transcription factors are simultaneously involved in regulation of *PPARγ*, *C/EBPα*, and *FABP4*, resulting in the inconsistent expression pattern with *MIR31HG*. Further study is needed to identify the possible mechanism.

LncRNAs can act as regulatory devices through combinations of modular functional components that include RNA-, DNA-, and protein-binding domains. They can create a scaffold for chromatin-modifying enzymes to epigenetically affect genomic loci^24^. For example, the lncRNAs *ANRIL* ^36^ and *HOTAIR* ^37, 38^ physically associate with a subunit of the polycomb repressive complex 2 to trimethylate H3K27 and specifically repress target genes. Similarly, the lncRNA *HOTTIP* binds WD repeat containing protein 5, a cofactor to histone methyltransferase MLL1 that mediates H3K4 trimethylation associated with gene activation^39^. A recent study has found the lncRNA *ADINR* regulates adipogenesis by transcriptional activation of *C/EBPα*. This lncRNA binds PA1 and recruits MLL3/4 complexes in the *C/EBPα* promoter to maintain H3K4me3 and remove H3K27me3 in these regions during adipogenic differentiation^16^. *MIR31HG* may exert its function by a similar mechanism. We have previously indicated several stable stem-loop structures in *MIR31HG* ^23^, which may provide the necessary spatial conformation for interaction with chromatin-modifying proteins. However, the modification complex recruited by *MIR31HG* to chromatin needs further investigation.

**Figure 8:**
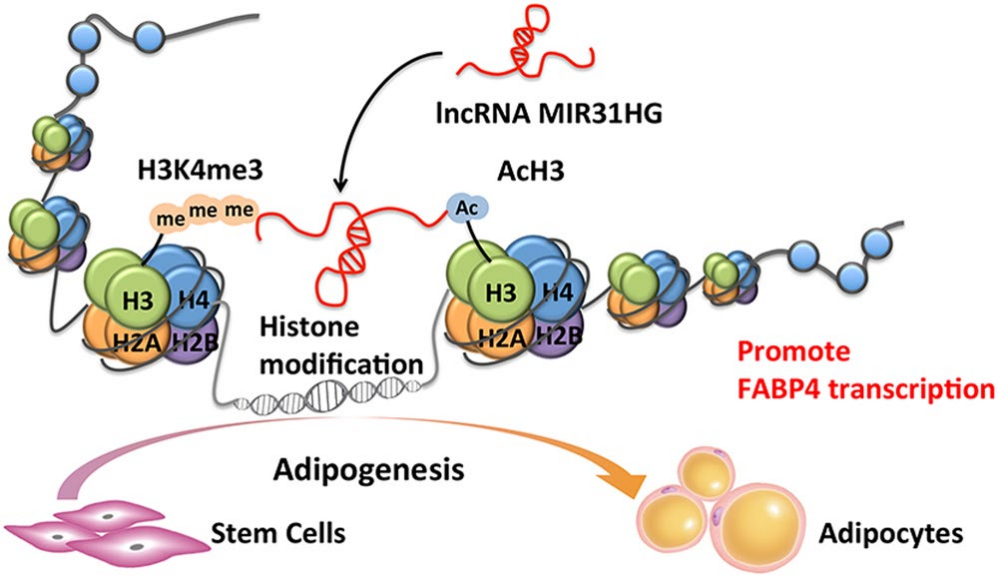
Schematics showing the regulation of adipogenesis by *MIR31HG*. *MIR31HG* promoted adipogenic differentiation of stem cells. In mechanism, *MIR31HG* contributed to histone H3 lysine 4 trimethylation and H3 acetylation at the *FABP4* gene locus and promoted its transcription, leading to the promotion of adipogenesis.

In conclusion, our study highlights the role of *MIR31HG* in adipocyte differentiation of hASCs. Knockdown of *MIR31HG* inhibits the adipocyte lineage commitment of stem cells. In mechanism, *MIR31HG* contributes to the enrichment of AcH3 and H3K4me3 in *FABP4* gene and promotes its transcription, leading to the promotion of adipogenesis (Fig. 8). Further research is needed to elucidate the potential use of *MIR31HG* as a molecular target for treating adiposity.

## Methods

### Cell culture

Primary hASCs from three different healthy human donors were obtained from ScienCell (San Diego, CA, USA) and their lot number were 2447, 8278, and 11537, respectively. The cells were cultured in growth medium (GM) consisting of DMEM with 10% fetal bovine serum and 1% antibiotics. All cell-based *in vitro* experiments were repeated in triplicate. For the adipocyte differentiation experiment, cells were allowed to become confluent for 1 day, and then cultured in standard growth medium supplemented with 10 μg/ml insulin (Sigma-Aldrich, St Louis, MO, USA), 100 nM dexamethasone (Sigma-Aldrich), 0.5 mM 3-isobutyl-1-methylxanthine (Sigma-Aldrich), and 200 μM indomethacin (Sigma-Aldrich). The adipogenic medium was changed every 2 days, and cells were harvested at the indicated times.

### Lentivirus infection

Recombinant lentiviruses containing full-length *MIR31HG* or the scrambled control (NC) were obtained from Integrated Biotech Solutions Co. (Ibsbio Co., Shanghai, China). Recombinant lentiviruses targeting *MIR31HG* (sh*MIR31HG*-1 and sh*MIR31HG*-2) or the scrambled nontargeting vector (shNC) were obtained from GenePharma Co. (Shanghai, China). Recombinant lentiviruses targeting *FABP4* (sh*FABP4*) was obtained from Integrated Biotech Solutions Co. Transfection of the hASCs was performed by exposing them to dilutions of the viral supernatant in the presence of polybrene (5 μg/mL) for 72 h, as described previously^23^. The recombinant lentivirus vectors contain the puromycin-resistance and GFP gene. 48 hours post transfection, puromycin was added to the cells at concentration of 10 µg/ml. The drug-resistant stable transfectants that express the gene of interest were selected. The transfection efficiency was then verified by fluorescence microscope tracking GFP, and the sorted cells were used as the stably expressing model in subsequent experiments.

### Cell viability analysis

The cell viability detection was performed using Cell Counting Kit-8 (CCK-8, Dojindo, Kumamoto, Japan) according to manufacturer’s instructions. Briefly, cells were seeded in 96-well plate (5 × 10^3^ cells/well). 10 ml of CCK-8 solution was added into each well at 24, 48, and 72 h after culture. Subsequently, the plates were further incubated for 1 h, then the absorbance was measured at 450 nm using a microplate spectrophotometer (Bio-Tek Instruments Inc., Winosski, VT).

### Oil red O staining

Oil red O staining was performed as described previously^40^. Cells were washed with phosphate-buffered saline (PBS) and fixed in 10% formalin for 30 min. The cells were subsequently rinsed with 60% isopropanol. Oil red O (0.3%, Sigma-Aldrich) was then added and incubated for 10 min with gentle agitation. After staining, the cells were washed with distilled water to eliminate unbound dye, visualized by light microscopy, and photographed. For quantitative assessment, Oil red O was eluted by 100% isopropanol and quantified by spectrophotometric absorbance at 520 nm against a blank (100% isopropanol).

### RNA isolation and qRT-PCR

Total RNA was extracted using TRIzol reagent (Invitrogen, Carlsbad, CA, USA) according to the manufacturer’s instructions and then reverse-transcribed into cDNA using the cDNA Reverse Transcription Kit from Applied Biosystems (Foster City, CA, USA). qRT-PCR was conducted with 1 μg of total RNA using the SYBR Green Master Mix on an ABI Prism 7500 real-time PCR System (Applied Biosystems) as described^41^. The following thermal settings were used: 95 °C for 10 min followed by 40 cycles of 95 °C for 15 s and 60 °C for 1 min. The primers used for *MIR31HG*, *PPARγ*, *C/EBPα*, *FABP4*, and glyceraldehyde 3-phosphate dehydrogenase (*GAPDH*, internal control for mRNAs and lncRNAs) are listed in Supplementary Table S1. The data were analyzed using the 2^−ΔΔCt^ relative expression method as described previously^41^.

### Western blot analysis

Western blot analysis was performed as described previously^41^. Briefly, cells were harvested, washed with PBS, and lysed in RIPA buffer. Proteins were separated by 10% sodium dodecyl sulfate–polyacrylamide gel electrophoresis and transferred to a polyvinylidene fluoride membrane by electroblotting. After blocking, proteins were detected by overnight incubation with primary antibodies against PPARγ (Cell Signaling Technology, Beverly, MA, USA), C/EBPα (HuaxingBio Science, Beijing, China), FABP4 (HuaxingBio Science), and GAPDH (Abcam, Cambridge, UK) at dilutions of 1:1,000. After washing, the membranes were incubated with secondary antibodies (Zhongshan Goldenbridge, Beijing, China, 1:10,000 dilution) at room temperature for 1 hour. The specific complexes were visualized using the ECL Kit (Applygen, Beijing, China). The intensities of the bands obtained by Western blot analysis were quantified using ImageJ software (http://rsb.info.nih.gov/ij/). The background was subtracted, and the signal of each target band was normalized to that of the GAPDH band.

### Fluorescent *in situ* hybridization (FISH)

*In situ* hybridization was performed with a Fluorescent *In Situ* Hybridization Kit (RiboBio, Guangzhou, China), as described previously^23^. Briefly, cells were fixed in 4% formaldehyde, permeabilized with 0.5% Triton X-100 for 5 min, and prehybridizated at 37 °C for 30 min before hybridization. Then an anti-*MIR31HG* oligodeoxynucleotide probe was used in the hybridization solution at 37 °C overnight in the dark. The next day, the cells were counterstained with DAPI and imaged using a confocal laser-scanning microscope (Carl Zeiss, Oberkochen, Germany).

### Cell fractionation

For the fractionation assay, cytoplasmic and nuclear RNAs were performed using a Nuclei Isolation Kit (KeyGEN, Nanjing, China), as described previously^23^. Briefly, cells were harvested and resuspended in lysis buffer. Then, the lysate was treated with Reagent A, incubated on ice for 15 min, followed by centrifugation at 4 °C. The pellet was then resuspended in lysis buffer followed by centrifugation. The supernatant was transferred to a new tube as the cytoplasmic fraction; the pellet was resuspended in Medium Buffer A and then added to a new tube with Medium Buffer B, followed by centrifugation at 4 °C. The supernatant was saved as the cytoplasmic fraction. The pellet was used as the nuclear fraction. RNA was extracted from both fractions using TRIzol.

### ChIP assay

ChIP assays were performed using the EZ-Magna ChIP assay kit (Merck Millipore, Darmstadt, Germany) according to the manufacturer’s instructions. Briefly, cells were washed with PBS and cross-linked with 1% formaldehyde for 10 min. Chromatin was sonicated on ice to generate chromatin fragments of 200–1000 bp, and then immunoprecipitated with each antibody. Antibodies for acetylated histone H3 (AcH3, Merck Millipore), tri-methylated histone H3K4 (H3K4me3, Merck Millipore), and isotype IgG (negative control, Cell Signaling Technology) were used in the immunoprecipitations. The immunoprecipitated materials were then washed extensively, and cross-linking was reversed. DNA from the eluted chromatin was purified by phenol extraction and ethanol precipitation. Input control DNA or immunoprecipitated DNA was determined by quantitative real-time PCR. Standard curves were constructed using a pool of input samples, and each ChIP sample was normalized to its respective input. We designed primers to separately amplify five regions in the *FABP4* promoter region. The primer pairs used for ChIP assays are shown in Supplementary Table S1.

### *In vivo* adipose tissue formation assay

The cells were adipogenically induced for 1 week before the *in vivo* study. Cell suspensions containing 5 × 10^6^ hASCs were incubated with Collagen Sponge (8 mm × 8 mm × 2 mm) for 2 h at 37 °C to allow the cells to attach to the scaffold. A total of 40 5-week-old BALB/c homozygous nude (nu/nu) mice were obtained from Charles River Laboratories (Beijing, China). The mice were maintained under specific pathogen-free conditions and randomized into four groups of 10 mice each: hASC/Collagen group (implanted with non-induced hASC/Collagen complex), Adi-hASC/Collagen group (implanted with adipogenic-induced hASC/Collagen complex), sh*MIR31HG*-hASC/Collagen group (implanted with adipogenic-induced sh*MIR31HG*-hASC/Collagen complex), and *MIR31HG*-hASC/Collagen group (implanted with adipogenic-induced *MIR31HG*-hASC/Collagen complex). The complex was implanted subcutaneously in the dorsum of the nude mice. All animal experiments were approved by the Peking University Animal Care and Use Committee (No. LA2014233). All experiments were performed in accordance with relevant guidelines and regulations.

### Histological observation

Neo-generated tissues were carefully dissected from the surrounding tissue 8 weeks after implantation. The harvested specimens were fixed in 4% paraformaldehyde and cut in half. One half was embedded in paraffin, and sectioned at 5 μm thickness for hematoxylin and eosin (H&E) staining, while the other half was frozen in Tissue-Tek OCT freezing medium (Sakura Finetek Inc., Torrance, CA, USA) and sectioned at 8 μm thickness for Oil red O staining.

### Quantification of newly generated adipose tissue

The proportion of the positive area for Oil red O was calculated by Lumina Visionâ (Mitani Corp., Tokyo, Japan) for quantification. Twenty fields at 40 × magnification in each microscopic photograph were chosen in a random fashion and the positive area was measured by the software. The percent square of the positive area was used as an indication of the degree of adipogenesis.

### Statistical analysis

Statistical analyses were performed using SPSS version 16.0 (SPSS, Chicago, IL, USA). All data are expressed as mean ± standard deviation (SD). Differences between groups were analyzed using Student’s *t*-test. In cases of multiple-group testing, one-way analysis of variance and post hoc Bonferroni test were adopted. A two-tailed value of *P* < 0.05 was considered statistically significant.

## Electronic supplementary material

Supplementary info
