# Supplementary material for: Knockdown of lncRNA MIR31HG inhibits adipocyte differentiation of human adipose-derived stem cells via histone modification of FABP4
Source: Sci Rep. 2017 Aug 14;7:8080. doi: 10.1038/s41598-017-08131-6 (PMC5556051; doi:10.1038/s41598-017-08131-6)
Supplement: Supplementary file 1 — Supplementary info [file 41598_2017_8131_MOESM1_ESM.pdf]

Knockdown of lncRNA *MIR31HG* inhibits adipocyte differentiation of human adipose-derived stem cells *via* histone modification of *FABP4*

Yiping Huang[1#], Chanyuan Jin[2#], Yunfei Zheng[1#], Xiaobei Li[1], Shan Zhang[3], Yixin Zhang[1], Lingfei Jia[3*], Weiran Li[1,4*]

[1]Department of Orthodontics, Peking University School and Hospital of Stomatology, Beijing 100081, China.

[2]Department of Prosthodontics, Peking University School and Hospital of Stomatology, Beijing 100081, China.

[3]Central Laboratory, Peking University School and Hospital of Stomatology, Beijing 100081, China.

[4]National Engineering Laboratory for Digital and Material Technology of Stomatology, Beijing Key Laboratory of Digital Stomatology, Beijing 100081, China

Supplementary Information

Supplementary Table S1. Sequences of RNA and DNA Oligonucleotides

Name	Sense Strand/Sense Primer (5'-3')	Antisense Strand/Antisense Primer (5'-3')
**Primers for qRT-PCR**		
MIR31HG	TCTCTGGTGCTTCCCTCCTT	GATCTAAGCTTGAGCCCCCA
PPARγ	GCTGTTATGGGTGAAACTCTG	ATAAGGTGGAGATGCAGGTTC
C/EBPα	GCAAGGCCAAGAAGTCGGTGGAC	TGCCCATGGCCTTGACCAAGGAG
FABP4	AGCACCATAACCTTAGATGGGG	CGTGGAAGTGACGCCTTTCA
GAPDH	GGTCACCAGGGCTGCTTTTA	GGATCTCGCTCCTGGAAGATG
**Primers for ChIP assay**		
Primer 1	AGGTAATGGAACCAGCAGC	CACTGTGAGGGTTGGAGCA
Primer 2	AAGTAGATTGACATAGCCACA	ACTTAGGTTGATTCCGTGA
Primer 3	CAGACGTACAGAGTGGCATAA	CCTCAGTCTCCCAAAGTGC
Primer 4	TCCAGAGTAGATGAGCGTTAT	AATGTTTGTGGCAGGAAAT
Primer 5	ATCTCCGAGGCAGTTCTTA	TTCTCCCTGGCAAATAGTC

Abbreviations: C/EBPα, CCAAT-enhancer-binding protein α; FABP4, fatty acid binding protein 4; GAPDH, Glyceraldehyde 3-phosphate dehydrogenase; PPARγ, peroxisome proliferator-activated receptor-γ.

Supplementary Figure 1

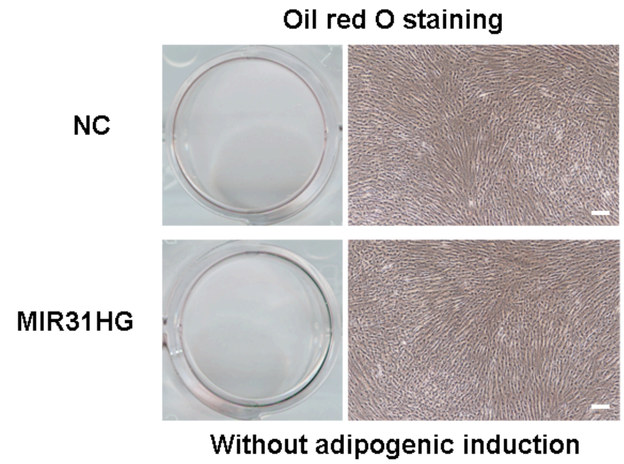

Supplementary Figure 1. Images of Oil red O staining in hASCs transfected with lentivirus expressing *MIR31HG* or the scrambled vector (NC) without adipocyte differentiation. Scale bar, 100 μm.
